# Supplementary material for: Atlas-scale single-cell DNA methylation profiling with sciMETv3
Source: Cell Genom. 2024 Dec 23;5(1):100726. doi: 10.1016/j.xgen.2024.100726 (PMC11770211; doi:10.1016/j.xgen.2024.100726)
Supplement: Document S1. Figures S1–S3 [file mmc1.pdf]

Cell Genomics, Volume *5*

Supplemental information

Atlas-scale single-cell DNA methylation profiling with sciMETv3

Ruth V. Nichols, Lauren E. Rylaarsdam, Brendan L. O'Connell, Zohar Shipony, Nika Iremadze, Sonia N. Acharya, and Andrew C. Adey

Atlas-scale Single-cell DNA Methylation Profiling with sciMETv3

Ruth V. Nichols[1,a], Lauren E. Rylaarsdam[1,a], Brendan L. O'Connell[1,2], Zohar Shipony[3], Nika Iremadze[3], Sonia N. Acharya[1], Andrew C. Adey[1,2,4,5,b]

1. Department of Molecular & Medical Genetics, Oregon Health & Science University, Portland, OR, USA
2. Cancer Early Detection Advanced Research Institute, Oregon Health & Science University, Portland, OR, USA
3. Ultima Genomics, Fremont, CA, USA
4. Knight Cardiovascular Institute, Oregon Health & Science University, Portland, OR, USA
5. Knight Cancer Institute, Oregon Health & Science University, Portland, OR, USA

[a] These authors contributed equally to this work
[b] Correspondence: adey@ohsu.edu

Supplementary Figures

Figure S1 sciMETv3 sequenced on the Illumina platform, related to Figure 3 2

Figure S2 sciMETv3 sequenced on the Ultima platform, related to Figure 3 3

Figure S3 sciMET+ATAC cell typing and filtering, related to Figure 5 4

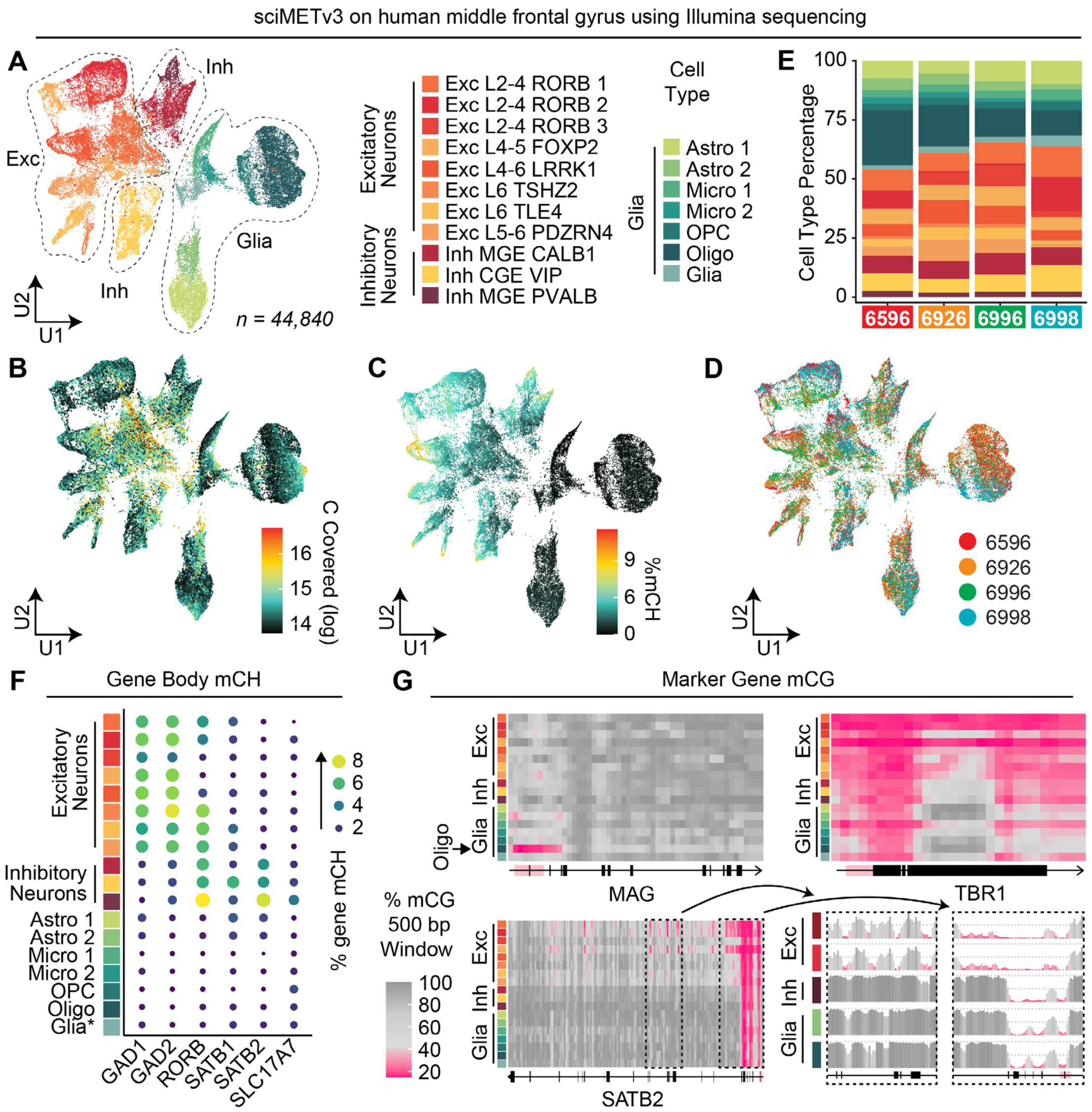

Figure S1 sciMETv3 sequenced on the Illumina platform, related to Figure 3

A) UMAP of cells colored by cell type. **B)** Cytosines covered per cell. **C)** Global mCH levels per cell. **D)** UMAP colored by individual. **E)** Cell type proportions by individual. **F)** Marker gene body mCH levels. **G)** Additional marker gene mCG methylation patterns with distinct cell type-specific hypomethylation regions.

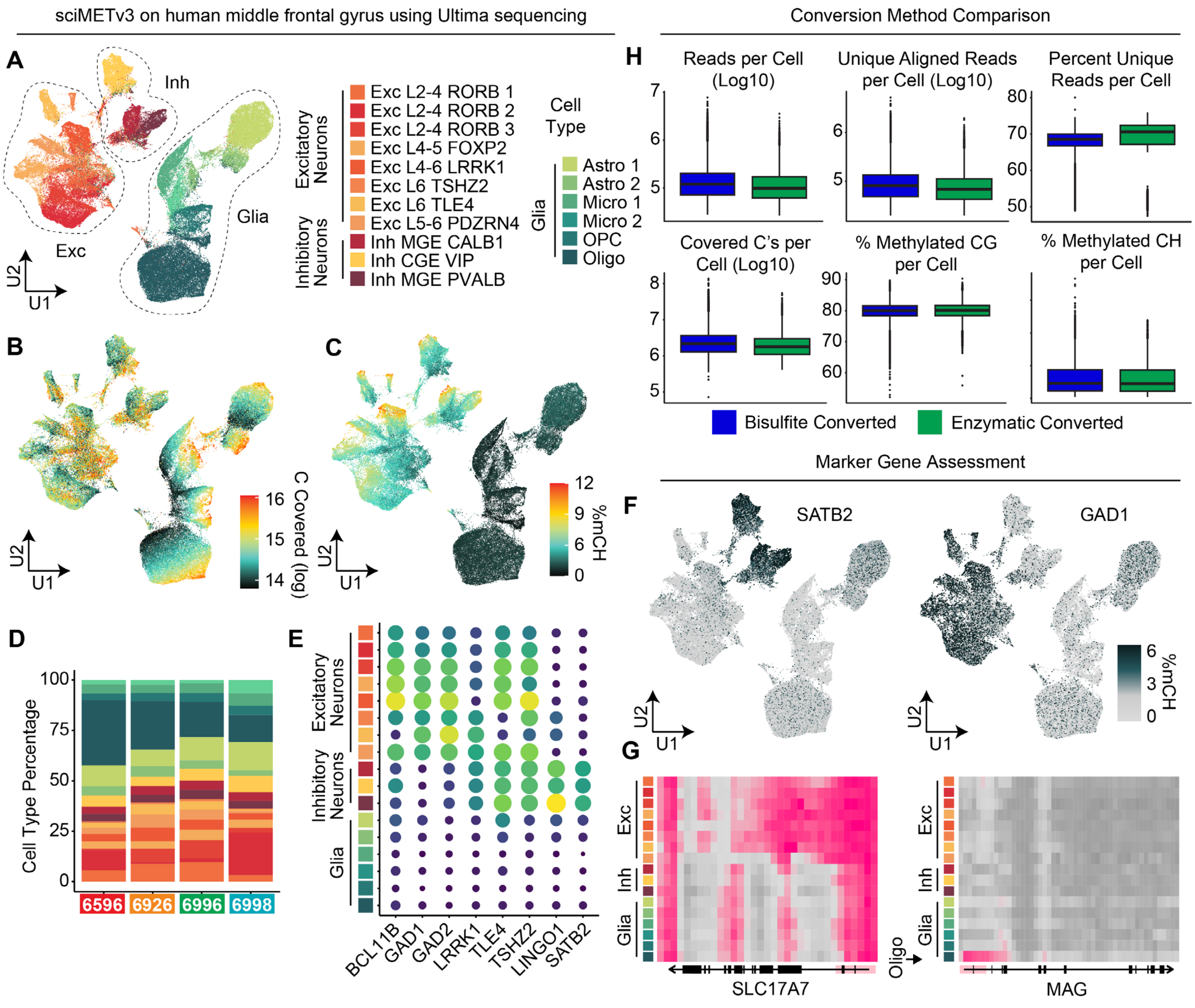

Figure S2 sciMETv3 sequenced on the Ultima platform, related to Figure 3

A) UMAP of cells colored by cell type. **B)** Cytosines covered per cell. **C)** Global mCH levels per cell. **D)** Cell type proportions by individual. **E)** Marker gene body mCH levels. **F)** mCH levels at the single-cell level projected onto the UMAP reveals inhibitory and excitatory neuron specificity. **G)** Additional marker gene mCG methylation patterns with distinct cell type-specific hypomethylation regions. **H)** Library performance statistics comparing bisulfite and enzymatic conversion methods.

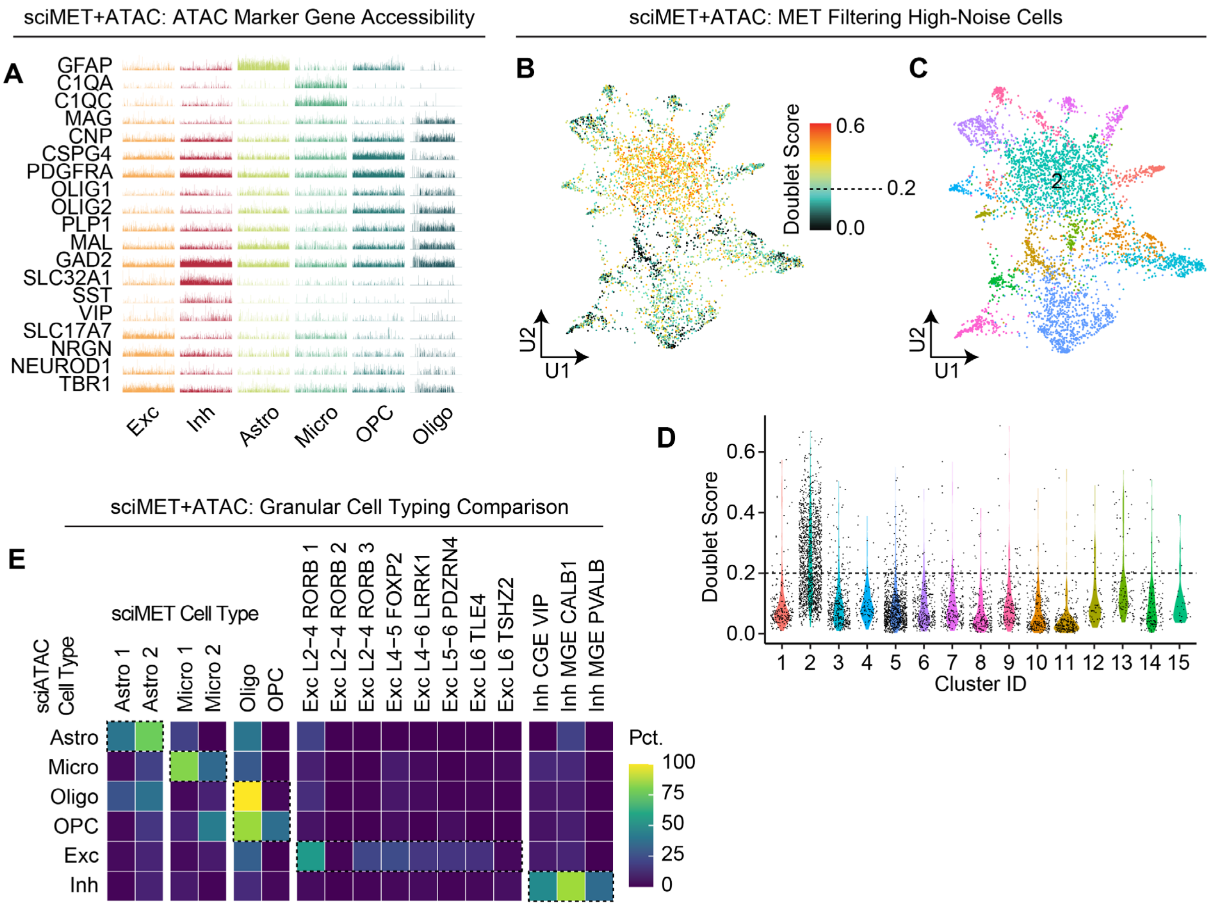

Figure S3 sciMET+ATAC cell typing and filtering, related to Figure 5

A) Marker gene tileplots for the ATAC modality. **B)** UMAP of sciMET+ATAC methylation cells reveals a population with a high doublet probability score. **C)** UMAP colored by cluster reveals that cluster 2 encompasses the high doublet score population. **D)** A score cutoff of 0.2 eliminates most of cluster 2 and other high-noise cells. **E)** Comparison of granular sciMET+ATAT DNA methylation-based cell types and ATAC-based cell types.
